# GLP-1/GLP-1R Signaling Regulates Ovarian PCOS-Associated Granulosa Cells Proliferation and Antiapoptosis by Modification of Forkhead Box Protein O1 Phosphorylation Sites

**DOI:** 10.1155/2020/1484321

**Published:** 2020-06-19

**Authors:** Zhihua Sun, Peiyi Li, Xiao Wang, Shuchang Lai, Hong Qiu, Zhi Chen, Shidi Hu, Jie Yao, Jie Shen

**Affiliations:** ^1^Endocrinology and Metabolism, The Third Affiliated Hospital of Southern Medical University, Guangzhou, Guangdong, China; ^2^Panyu Central Hospital, Guangzhou, Guangdong, China; ^3^Medical Research Center, Shunde Hospital of Southern Medical University, Shunde, Guangdong, China

## Abstract

As the major cause of female anovulatory infertility, polycystic ovary syndrome (PCOS) affects a great proportion of women at childbearing age. Although glucagon-like peptide 1 receptor agonists (GLP-IRAs) show therapeutic effects for PCOS, its target and underlying mechanism remains elusive. In the present study, we identified that, both *in vivo* and *in vitro*, GLP-1 functioned as the regulator of proliferation and antiapoptosis of MGCs of follicle in PCOS mouse ovary. Furthermore, forkhead box protein O1 (FoxO1) plays an important role in the courses. Regarding the importance of granulosa cells (GCs) in oocyte development and function, the results from the current study could provide a more detailed illustration on the already known beneficial effects of GLP-1RAs on PCOS and support the future efforts to develop more efficient GLP-1RAs for PCOS treatment.

## 1. Introduction

Polycystic ovary syndrome (PCOS) is diagnosed in 6%–8% of women at childbearing age [1]. The manifestations of PCOS generally include oligomenorrhea or amenorrhea, abnormal folliculogenesis, chronic anovulation, hyperandrogenemia, and infertility [2]. Other than being the most frequent cause of female anovulatory infertility [3], the prevalence of metabolic syndrome, dyslipidemia, and type 2 diabetes is much higher in PCOS women compared to normal age and body mass index matched women. PCOS is a severe menace to the physical and mental health of patients.

The complex pathogenesis of PCOS includes abnormal expression of apoptosis correlated genes, MAPK signaling pathway, and cell cycle associated molecules [4]. More specifically, the proliferation and apoptosis of GCs are the fundamental causes of follicular development and atresia [5]. Taken these together, the study of PCOS GCs is helpful to investigate its physiological and pathological roles during disease progression and further possible pathogenesis.

Glucagon-like peptide 1 (GLP-1) is a hormone produced during the processing of proglucagon by the intestinal epithelial endocrine L-cells. In healthy individuals, GLP-1 is the major incretin hormone [6], and GLP-1 (7-36) Acetate is the bioactive form of GLP-1. GLP-1 acts through the GLP-1 receptor (GLP-1R), which is a class B G-protein-coupled receptor. Considering its reported effects on insulin, the GLP-1/GLP-1R axis is a promising drug target for metabolic diseases [7, 8]. In addition to the effects of GLP-1/GLP-1R axis on metabolism, GLP-1R agonists (GLP-1RAs, such as liraglutide) also improved the markers of ovarian function (bleeding pattern; levels of AMH (Anti-Müllerian hormone)), sex hormones, gonadotropins, and ovarian morphology in overweight women with PCOS [9]. Moreover, preconception intervention with low-dose liraglutide added to metformin increased the *in vitro* fertilization pregnancy rate in infertile obese women with PCOS [10]. With the GLP-1RAs treatment, it was also observed that the level of androgens was modestly decreased and the menstrual frequency was increased [8, 11, 12]. Some scholars think that GLP-1 might be one of the most important modulating signals connecting the reproductive and metabolic system and there is mostly stimulating role of GLP-1 and its mimetics in mammalian reproduction that goes beyond mere weight reduction [13]. However, understanding of the role of GLP-1 and GLP-1RAs in reproduction is currently limited. The beneficial therapeutic effects on PCOS ovarian functions could be caused by the improvement in metabolism after GLP-1RA treatment or the direct effects of the drugs on ovaries.

GLP-1R is expressed in numerous tissues, including the pancreas, kidney, heart, lung, adipose tissue, and smooth muscle, as well as in specific regions of the central nervous system. The wide distribution of GLP-1R suggests that GLP-1 has independent effects on different tissues [6]. In contrast, few studies have been conducted focusing on GLP-1R expression in the ovary with an exception that reported the GLP-1R is expressed in human ovarian tumor cells [14]. Our previous study (Figure S1) identified the presence of GLP-1R on the membrane and cytoplasm of mouse ovarian granulosa cells. Thus, our central hypothesis is that GLP-1/GLP-1R plays an essential role in mediating the function of MGCs.

FoxO1, a member of the class O in Forkhead transcription factor (Fox) family, regulated by PI3K/Akt pathway, is involved in many pathological and physiological processes including cell proliferation, apoptosis, autophagy, metabolism, inflammatory response, and so forth [15]. FoxO1 gene is expressed in GCs of follicles at different developmental stages, and FoxO1 expression is the highest in GCs of atresia follicles, mainly in the nucleus of GCs [16, 17]. Most importantly, FoxO1 plays an important role in promoting follicular atresia and apoptosis of GCs of PCOS [18]. Interaction between GLP-1 and FoxO1 has been reported in pancreatic B cells [19, 20]. GLP-1 can activate the PI3K pathway, increase FoxO1 transfer out of the nucleus, induce the target genes pdk-1 and FoxA2 of FoxO1, so as to promote the proliferation and antiapoptosis of B cells. However, there are few studies on the interaction between GLP-1 and FoxO1 in GCs. In this present study, a mouse model of PCOS was established to mimic the serological and pathological alterations of the disease. Based on previous results, the next step of the current study was to investigate whether GLP-1 regulates ovarian PCOS-associated MGCs proliferation and antiapoptosis by modifying forkhead box protein O1 phosphorylation sites.

## 2. Material and Methods

### 2.1. PCOS Mouse Model and Treatment

A total of 50 three-week-old female C57BL6 mice (body weight 8.85 ± 10.42 g), which were selected and weaned at 21 days of age, were provided by the Guangdong Provincial Animal Experimental Center. All animals were bred, housed at 25°C (humidity 50%), and were acclimated to standard laboratory conditions (12 h light and 12 h dark cycle) with free access to rodent feed and water. After 2 days of adaptive feeding, mice were randomly divided into two groups (vehicle group (*n* = 10), DHEA group (*n* = 40)). The mice in the vehicle group were injected daily with sesame oil (Sigma, MO, USA, 0.1 mL/100 g) while the mice in the DHEA group were injected with dehydroepiandrosterone (DHEA, Sigma, 6 mg/100 g·d) [21] subcutaneously daily for 20 consecutive days.

At 32 days of age, a subgroup of the DHEA treated mice (*n* = 10) received twice daily injected (s.c.) with liraglutide (Novo Nordisk 0.2 mg/kg) [22] for 21 consecutive days and the rest (*n* = 30) also received saline injections twice daily. Between 6 and 7 weeks, daily vaginal cytology was measured every morning in the three groups, DHEA + liraglutide, DHEA, and vehicle. Vaginal cells were collected using saline lavage, fixed with methanol and stained with Wright staining. The cycle of vaginal exfoliative cells was observed under a microscope for seven consecutive days, and the stage of the estrus cycle was assessed and recorded. The PCOS model was considered to be successfully established for further experiments until the lost in the estrus cycle and the predominance of the cornified squamous epithelial cells were observed in the DHEA group. The weights of all mice were measured every two days throughout the experiment. The animal experiments were performed in accordance with the Southern Medical University Institutional Animal Care and Use Committee Policies for Animal Use under an approved animal protocol.

### 2.2. Tissue Collection and Histology

After the treatment with liraglutide (day 54 of life), both reproductive and metabolic features were evaluated. All mice in the three groups went under fasting conditions after performing a vaginal smear test indicating that they were on estrus or proestrus stage. All mice were weighed after fasting for 8 h and each given 4% chloral hydrate intraperitoneally to anesthetize. The whole blood was kept at room temperature for 1 hours and centrifuged at 1,000 g for 15 min at 4°C. The serum was separated and stored at –80°C for glucose, insulin, and testosterone measurement. Serum insulin and testosterone were determined using enzyme-linked immunosorbent assay kits (ARG81295, ARG80662, Arigo, Taiwan). After the mice were sacrificed, the bilateral ovaries were checked and quickly collected. Following the collection, ovaries isolated from ten mice of each group were fixed with 10% formalin at 4°C for 8 hours. The fixed tissues were dehydrated in a graded ethanol series, cleared in xylene, and embedded in paraffin wax. The 3 to 4 *μ*m sections were routinely cut, affixed to siliconized glass, dehydrated, and then subjected to hematoxylin and eosin (H&E) staining for histomorphological analyzation. While the other ovaries from twenty PCOS mice were used MGCs isolation purpose.

### 2.3. Isolation of Mouse Ovarian Granulosa Cells and Culture

The PCOS mice were used for isolating ovarian MGCs. After the sacrifice of the mice, the ovaries were collected and cultured as follows. The ovaries were washed with Hank's equilibrium solution and transferred to a fresh DMEM (Dulbecco's Modified Eagle's medium) F12 medium (containing 10% fetal bovine serum (FBS)). The presinusoidal and sinusoidal follicles were pierced with a 25-gauge needle. The oocytes and MGCs are isolated from the remaining ovarian tissue and released into the medium. Subsequently, the cell suspension was filtered with a 40 μm cell strainer, and MGCs were collected by centrifugation at 300*g* for 5 min at 4°C. The MGCs viability was determined by trypan blue exclusion, and the cells were then suspended and cultured in DMEM containing 10% FBS, 1 mM pyruvate, 2 mM glutamine, 100 IU/mL penicillin, and 100 mg/mL streptomycin. The seeded cells were cultured at 37°C and 5% CO_2_ for 24 h. Immunofluorescence staining was used to detect the expression and localization of FSHR (follicle-stimulating hormone receptor), a specific marker of GCs, to identify the isolated cells as GCs [23].

### 2.4. Immunofluorescence

The MGCs isolated from the PCOS mice ovaries were fixed in 4% formaldehyde diluted for 15 mins at room temperature, rinsed three times in PBS, and were blocked in 5% Normal Goat Serum/0.2% Triton for 60 min. After aspirating blocking solution, the cells were incubated in a moisturized staining box with rabbit anti-FSHR polyclonal antibody (Shanghai Kalang, Shanghai, China) overnight at 4°C. After primary antibody incubation, the cells were washed three times, 5 min each, with PBS. Following the wash, the cells were incubated in dark with an appropriate secondary antibody of designed dilution at room temperature for 1 h. After decanting the secondary antibody solution, the cells were washed three times with PBS, 5 min each. Lastly, the cells were incubated with 1 mg/mL DAPI (4′,6-diamidino-2-phenylindole) for 1 min at room temperature and rinsed once with PBS. After sealing, images were taken and obtained under a laser confocal microscope.

### 2.5. Construction and Transfection of the FoxO1⁃Ser 256 Mutant Lentivirus Plasmid Vector

To further clarify whether GLP-1 regulates ovarian PCOS-associated MGCs proliferation and apoptosis by modification of forkhead box protein O1 phosphorylation sites, we construct the lentivirus vector expressing FoxO1-Ser 256 phosphorylation site mutant (FoxO1-mt): Lv201-FoxO1 (S256A)-Sv40-EGFP-IRES-Puro and FoxO1 wild type lentivirus vector (FoxO1-wt): Lv201-FoxO1-Sv40-EGFP-IRES-Puro. Lv201 was purchased from GeneCopoeia. The PCOS MGCs were seeded into six-well plates (1 × 10^6^/mL) and incubated in DMEM-F12 medium (containing 10% FBS) for 24 h. The cells were randomly divided into four groups: blank cell control group, vector group, FoxO1⁃wt group, and FoxO1-mt (S256A) group. The appropriate viral concentration gradient was determined by a previous pilot study. Each well was added with 5 *μ*l virus suspension. The plates were placed in the incubator and cultured at 37°C. After changing the medium at 12 hours of transfection, the cells were cultured continuously. After 72 hours, the transfection efficiency was assessed under a fluorescence microscope and used for subsequent experiments.

### 2.6. Measurement of Cell Viability (CCK-8 Assay)

The PCOS MGCs were used to prepare a single-cell mixture. The cell density was adjusted to 1 × 10^5^/mL, and the cells were seeded into 96-well plates. Each well contained 100 *μ*L of cell suspension, and six replicate wells were set up for routine culture. After the cells reached 80% confluency, 10 *μ*L of stimulated containing different concentrations of GLP-1 (7-36) (HY-P0054, MedChemExpress, NJ, USA) (0, 10, 100, 1000 nmol/L) were added to the cells for 24, 48, 72, 96, and 120 h. Subsequently, 10 *μ*L of cell counting kit-8 (CCK8) solution was added to each well and incubated at 37°C for 2 h. The absorbance value OD was measured with a microplate reader at a wavelength of 450 nm, and the cell viability was calculated accordingly. The experiment was repeated thrice.

### 2.7. Flow Cytometry

The PCOS MGCs were seeded in six-well plates (1 × 10^6^/mL) and cultured with DMEM-F12 medium (containing 10% FBS) for 24 in triplicate. After treatment with gradient concentrations of GLP-1 (7-36) (0, 10, 100, 1000 nmol/L) as indicated in the figure legends, the cells were maintained in culture for 48 h. At the end point, the cells were harvested and washed thrice with PBS. After centrifuging at 1000 rpm for 5 min, Annexin V-FITC staining and PI double staining were performed. Apoptosis rate was assessed using a FACS analyzer (Becton Dicknson, San Jose, CA, USA). Annexin V-FITC-positive were evaluated as apoptotic or late-apoptotic.

### 2.8. Western Blotting

The PCOS MGCs were seeded in 21 cm^2^ cell culture dishes at a density of 1 × 10^6^/well and incubated for 16 h to ensure cell attachment. The cells were continuously cultured in DMEM-F12 medium (containing10% FBS) for 24 h. After treatment with 100 nM GLP-1 (7-36) as indicated in the figure legends, the cells were maintained in culture for 48 h. Samples were harvested using RIPA lysis buffer (1% Triton X-100, 0.1% SDS, 1 mM PMSF, 1 *μ*g/mL aprotinin, and 1 *μ*g/mL leupeptin in PBS). Protein concentrations were determined by BCA assay (Beyotime). About 40 *μ*g of total protein were electrophoresed on SDS-PAGE gels and transferred to PVDF membranes (Millipore). The membranes were blocked in Tris-buffered saline (TBS) containing 0.1% Triton X-100 (TBS-T) and 5% (w/v) BSA for 90 min at room temperature. The membranes were then incubated overnight at 4°C with primary antibodies diluted in TBS-T containing 5% (w/v) BSA as follows: GLP-1R (1 : 500) (number ARG65819, Arigo), bcl-2 (1 : 1000) (number A16776, ABcolnal), bax (1 : 1000) (number #2772, CST), FoxO1 (1 : 500) (number A13862, ABcolnal), pFoxO1 (Ser 391) (1 : 500) (number AP0176, ABcolnal), and pFoxO1 (Ser 256) (1 : 500) (number #9461, CST). After washing primary antibody, the membranes were incubated for 1 h with secondary antibodies at 1 : 2000 dilution. The specific reaction band was visualized using the enhanced chemiluminescence system and exposed. Finally, the ImageJ (MD, USA) image analysis software was used to determine the electrophoretic band gray value. The operation after transfection was the same as above.

### 2.9. Statistical Analysis

Quantitative data were presented as the means ± standard error of mean (SEM) of three independent experiments. Statistical analyses were performed with the GraphPad Prism 7.0 (La Jolla, CA, USA) software. Pairs of groups were compared with two-tailed *t* tests for paired or unpaired data. For comparisons of more than two groups, significance was determined by an analysis of variance (ANOVA). A *P* value less than 0.05 was considered statistically significant.

## 3. Results

### 3.1. Liraglutide Promoted the Granulosa Cells Proliferation of DHEA-Induced PCOS Mouse

*In Vivo* and Recovered Their Regular Estrous Cycles Partially

**Figure 1:**
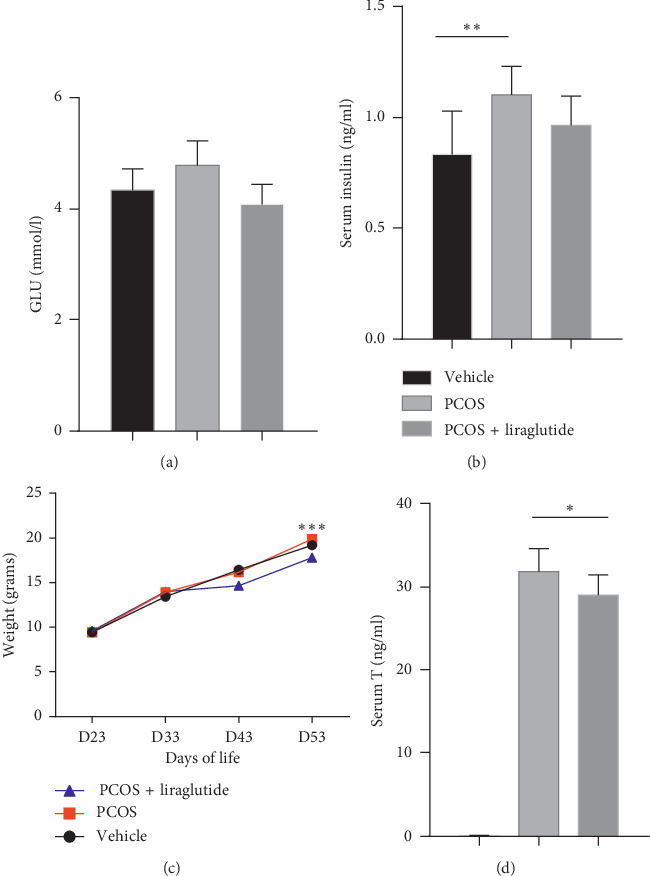
Serological and metabolic features were altered with or without treatment of liraglutide in PCOS model mice compared with vehicle group. The fasting blood glucose (a), serum insulin level (b), and blood testosterone level (c) were measured in serum samples collected from the three groups, PCOS + liraglutide, PCOS, and vehicle on D54 of life. The body weight was measured on D23, D33, D43, and D53 of life (d). The data were expressed as mean ± SEM (*N* = 10 mice/one group). ^*∗*^*P* < 0.05, ^*∗∗*^*P* < 0.01, and ^*∗∗∗*^*P* < 0.005.

A mouse model of PCOS was established by injecting dehydroepiandrosterone (DHEA) to explore the GLP-1/GLP-1R signal in PCOS ovaries. The results of continuous vaginal smear monitoring showed that all mice in the vehicle group had a regular estrous cycle of 4 to 5 days. After the treatment, vaginal smears revealed that 4 mice (40%) in the liraglutide group recovered to regular estrous cycles, while those in the PCOS group still maintained the absence of the estrus cycle. In agreement with the metabolic disorder frequently observed in PCOS, the fasting insulin levels in mice (day 54 of life) with PCOS increased compared with the mice in the vehicle group (*P* < 0.05) (Figure 1(b)). Fasting blood glucose levels were also higher in the PCOS group than that in the vehicle group, although the effect was marginal (Figure 1(a)). Despite the abnormal metabolism, the body weight of mice in the PCOS group was not different from vehicle control group (Figure 1(c)). Moreover, as a major hormone in female reproduction process, testosterone levels increased more than 300-fold in PCOS mice (day 54 of life) (*P* < 0.01) (Figure 1(d)). As shown in the figure, the intervention of liraglutide significantly reduced the body weight of PCOS mice (*P* < 0.01). It is also observed that the fasting insulin of mice in the liraglutide group was lower than that in the PCOS group, however, not statistically significant. Similarly, testosterone levels after liraglutide treatment slightly decreased compared with the mice in the PCOS group (*P* < 0.05).

**Figure 2:**
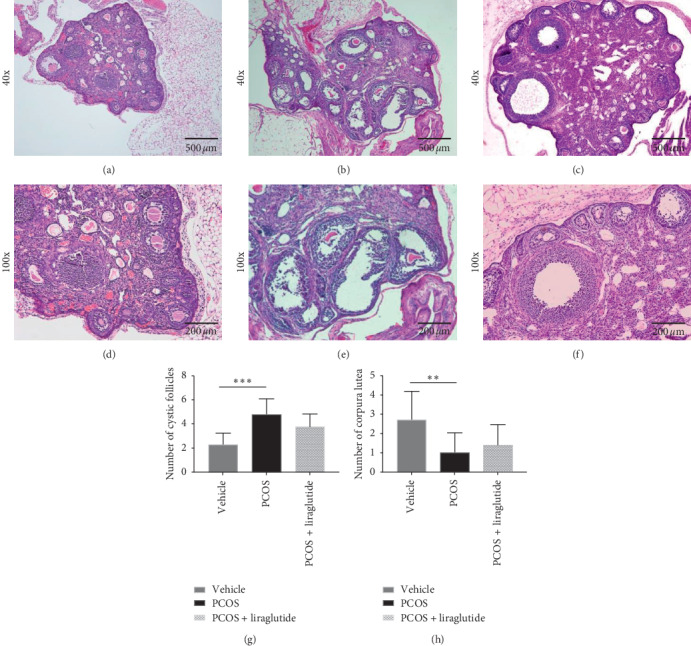
Ovarian histopathology changed in PCOS model mice with or without treatment of liraglutide. H&E staining of thin sections of ovaries from mice of three groups, PCOS + liraglutide (c, f), PCOS (b, e), and vehicle (a, d). Micrographs were taken at magnifications: ×40, ×100, and scale bars represent 500 *μ*m and 200 *μ*m, respectively. The numbers of cystic follicles (g) and corpora lutea (h) were counted based on the images after staining. The data were expressed as mean ± SEM (*N* = 10/group). ^*∗*^*P* < 0.05, ^*∗∗*^*P* < 0.01, and ^*∗∗∗*^*P* < 0.005.

To further characterize the disease progression in PCOS mice, the morphological changes of ovaries were examined. The overall ovarian volume increased along with the observation of pale color in PCOS mice. Mice with PCOS also displayed polycystic changes, cystic distended follicles, and multiple atretic follicles (Figures 2(b) and 2(e)). Additionally, the MGCs thickness reduced to two to three layers and was loosely arranged with loss of oocyte or radiation corona in the vesicles; moreover, the luteum formation was rarely seen in mice with PCOS (Figures 2(b) and 2(e)). The number of antral follicles (*d* > 300 *μ*m, per ovary) increased in the PCOS group compared with the control (*P* < 0.05) (Figure 2(g)). All these pathological observations were in accordance with the syndromes in PCOS patients in the clinic and were not observed in normal control mice (Figures 2(a) and 2(d)). After treatment with liraglutide, the number of layers of MGCs in the follicles of PCOS mice increased significantly, cystic dilated follicles and multiple atresia follicles decreased, and corpus luteum was visible (Figures 2(c) and 2(f)).

### 3.2. Effects of GLP-1 on Isolated Granulosa Cells from PCOS Mouse Ovary Survival

Given the distribution of GLP-1R in MGCs, the GLP-1/GLP-1R axis might regulate ovarian function via controlling the proliferation of MGCs. Taken together, the proliferation effects of GLP-1 (7-36) on primary MGCs were examined.

Primary PCOS-associated MGCs showed monolayer adherence within 24 hours; however, the growth rate *in vitro* was slower. On the third day of culture, the adhered cells reached the proliferation plateau stage with high viability. The cultured cells showed complete morphology, spindle or star shape, clear margin, uniform size, large, round nucleus, good transparency of cytoplasm, and rich granules (Supplementary Figure S2). On the 6-7th days of culture, the cells began to deform and went under apoptosis due to over confluency.

**Figure 3:**
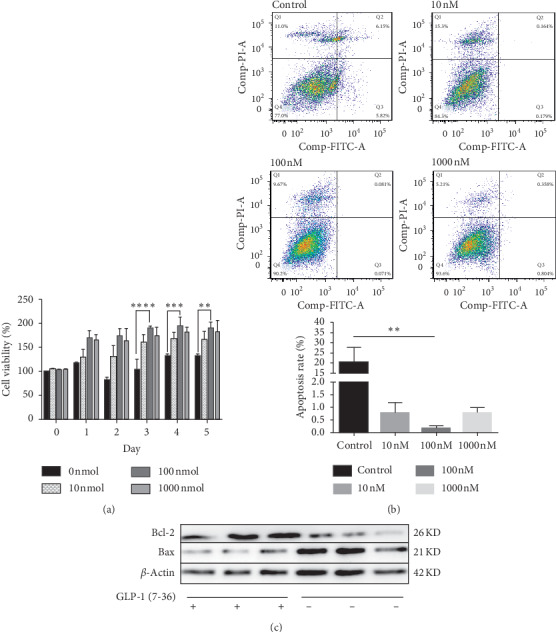
Proliferation and antiapoptosis of granulosa cells of PCOS mice were promoted by GLP-1 (7-36) *in vitro*. (a) GCs were isolated from the ovaries of PCOS model mice (day 54 of life) and treated with increasing doses of GLP-1 (7-36) (0, 10, 100, and 1000 nM). Cell viability was measured by CCK-8 assay. (b) Flow cytometry Annexin V-FITC/PI double staining was used to detect the apoptotic rate of PCOS MGCs in control group and GLP-1 (7-36) (10 nM, 100 nM, and 1000 nM) treated for 48 hours. (c) Bcl-2, bax, and beta-actin were detected using their specific antibodies by the Western blot analysis for the PCOS MGCs with or without 100 nM GLP-1 (7-36) interference for 48 hours. Results are expressed in at least three separate experiments. ^*∗*^*P* < 0.05, and ^*∗∗*^*P* < 0.01.

The MGCs with PCOS were stimulated with GLP-1 (7-36) at different concentrations (0, 10, 100, 1000 nM). Cell viability was determined by the CCK8 assay, which measured the OD (optical density) value of the samples. The results (Figure 3(a)) showed that 100 nM GLP-1 (7-36) significantly enhanced the viability of MGCs compared with the blank control group. Thus, in subsequent experiments, we investigated the mode of action of GLP-1 (7-36) at a concentration of 100 nM.

By flow cytometry (Figure 3(b)), we found that different concentrations of GLP-1 (7-36) could inhibit the apoptosis of PCOS-associated MGCs treated by DHEA. After being treated for 48 hours, the percentage of apoptotic and late-apoptotic cells was 11.97%, 0.343%, 0.152%, and 1.162%, corresponding to the concentration of GLP-1 (7-36) of 0, 10, 100, and 1000 nM. Similarly, the expression of antiapoptotic protein bcl-2 and proapoptotic protein Bax in PCOS ovarian MGCs treated with 100 nM GLP-1 for 48 hours was detected by Western blot. The results showed that the expression of bcl-2 protein increased significantly after GLP-1 intervention, while the expression of Bax decreased (Figure 3(c)).

### 3.3. The Effect of GLP-1 (7-36) on Phosphorylation Modification of FoxO1

**Figure 4:**
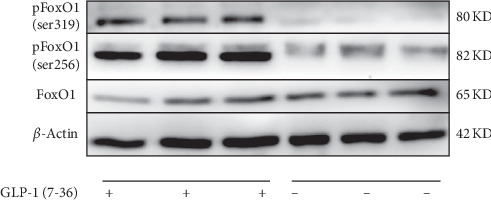
GLP-1 (7-36) induced phosphorylation of FoxO1 in PCOS ovarian MGCs. The isolated PCOS mice ovarian GCs were pretreated in the presence or absence of 100 nM GLP-1 (7-36) to detect FoxO1, pFoxO1 (Ser 256), and pFoxO1 (ser 391) using their specific antibodies by the Western blot analysis. The experiment was repeated thrice.

Given that phosphorylation modification is an important regulator of FoxO1 transcriptional activity [24]. We detected the changes of FoxO1 and pFoxO1 in PCOS-associated MGCs treated with or without 100 nM GLP-1 (7-36) for 48 h by Western blot. As shown in Figure 4, FoxO1 and pFoxO1 (Ser 256 and Ser 391) signaling was detected in PCOS-associated MGCs. We observed that the expression of FoxO1 protein in the GLP-1 (7-36) intervention group was not notably increased compared to the control group. We also found that the level of FoxO1 protein phosphorylation in the sites of Ser 256 and Ser 319 was significantly upregulated after GLP-1 (7-36) treatment. Therefore, we concluded that GLP-1 (7-36) significantly increased FoxO1 phosphorylation without inducing the expression of FoxO1 in DHEA-induced PCOS ovarian MGCs.

### 3.4. The Inhibitory Effect of GLP-1 (7-36) on the Apoptosis of DHEA-Induced PCOS Ovarian Granulosa Cells Depends on FoxO1 Protein Phosphorylation Modification

**Figure 5:**
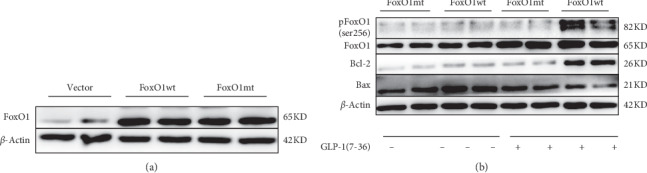
GLP-1 (7-36) protected PCOS MGCs by modification of forkhead box protein O1 phosphorylation sites. (a) FoxO1 was detected using their specific antibodies by the Western blot analysis for the PCOS MGCs after transfection. (b) FoxO1, pFoxO1 (ser 256), bcl-2, and bax were detected using their specific antibodies by the Western blot analysis for the PCOS MGCs treated with 100 nM GLP-1 (7-36) for 48 h after transfection as indicated in the figure legends. The experiment was repeated thrice.

Primary MGCs derived from DHEA-induced PCOS mice were transfected with FoxO1-wt, FoxO1-mt, and vector group and the transfection rate exceeded 90%. Western blot results confirmed the overexpression of FoxO1 in MGCs after transfection. The expression level of FoxO1 protein in the FoxO1-wt group and FoxO1-mt group was similar (Figure 5(a)). Therefore, on the premise of ensuring that the expression level of FoxO1 is consistent, we explored whether GLP-1 (7-36) could regulate DHEA-induced PCOS ovarian MGCs proliferation and apoptosis by modifying FoxO1 phosphorylation site. As shown in Figure 5(b), a significant increase in FoxO1 phosphorylation of the FoxO1-wt group was noted, as well as no obvious effects of GLP-1 (7-36) on the phosphorylation of FoxO1 in the presence of FoxO1-mt. pFoxO1 protein level of the -wt group with GLP-1 intervention was significantly enhanced compared to that without intervention. These data indicated that GLP-1 (7-36) could modulate the FoxO1/pFoxO1 signaling pathway.

To assess whether GLP-1-induced PCOS-associated MGCs antiapoptotic was dependent on FoxO1 protein phosphorylation modification, the ovarian GCs of PCOS mice were cultured with or without 100 nM GLP-1 (7-36) for 48 h after transfection. The cultured cells were then assessed using Western blotting (Figure 5(b)). The expressions of bcl-2 and bax in MGCs of PCOS mice treated with GLP-1 (7-36) after transfection were detected. The expression of bcl-2 protein increased significantly with GLP-1 (7-36) intervention after FoxO1-wt transfection; in the contrary, the expression of Bax protein decreased. However, there was no significant difference in the FoxO1-mt transfection group before and after GLP-1 (7-36) intervention. Therefore, GLP-1 (7-36) could inhibit GCs apoptosis in PCOS mice by modification of FoxO1 protein phosphorylation sites.

Taken together, these results suggest that the phosphorylation of FoxO1 is associated with the protective effects of GLP-1 on PCOS-associated ovarian MGCs.

## 4. Discussion

PCOS is the most frequent cause of female anovulatory infertility [3]. It is accompanied by a series of metabolic disorders that may disrupt ovarian function, such as dyslipidemia, type 2 diabetes, insulin resistance, and compensatory hyperinsulinemia [25]. The hormone peptide or the agonists of the hormone receptors have shown efficient therapeutic effects in clinical practice. Other than their positive effects on metabolism, GLP-1RAs (such as liraglutide) also improved ovarian function in women with PCOS. But the remaining important question is that the underlying mechanism of GLP-1/GLP-1R axis on ovaries is not clearly illustrated.

In this study, a mouse model of PCOS was established to address the aforementioned issue. After treating PCOS mice with liraglutide, we conclude that liraglutide could effectively improve ovarian polycystic dilatation, promote GCs proliferation, and facilitate follicular development. As we identified previously, the GLP-1R was presented on the membrane and cytoplasm of mouse ovarian granulosa cells and the expression of GLP-1R is decreased in the PCOS model (Supplementary Figure S1). From there, we hypothesized that GLP-1/GLP-1R plays an essential role in mediating the function of ovarian GCs of PCOS mice. To further test our hypothesis, we investigated the effects of GLP-1 (7-36) on ovarian GCs proliferation and apoptosis of PCOS mice *in vitro*, along with the underlying molecular mechanism.

During follicular development, GCs secrete follicular fluid, steroid hormones, and growth factors. The secretion regulates the growth, differentiation, and maturation of follicular cells and oocytes, thereby regulating the development of follicles [26]. The dysfunction in GCs resulted in abnormal follicular development. Also, GCs apoptosis-induced follicular atresia is the main cause of failure in dominant follicle selection [27]. In DHEA-induced PCOS mice model, the number of apoptotic cells in preantral and antral follicles increased, and the expression of apoptotic protein in GCs with TUNEL-positive follicles was stronger than that with TUNEL-negative follicles. These results, all together, suggested that GCs apoptosis played an essential role in the mediation of follicular atresia in PCOS [28]. GLP-1 is an incretin hormone that exhibits several pharmacological actions such as neuroprotection, increased cognitive function, cardioprotection, decreased hypertension, suppression of acid secretion, and protection from inflammation [29]. GLP-1 can act through its receptor (GLP-1R) on stimulating cells proliferation and inhibiting cells apoptosis [19, 30–33]. Our data interpretation suggested the direct action of GLP-1/GLP-1R axis on MGCs to modulate PCOS pathogenesis. Meanwhile, we confirmed that GLP-1 (7-36) significantly attenuated PCOS-associated ovarian MGCs apoptosis in a concentration-dependent manner.

FoxO1 protein is a negative regulator of cell survival. Its main function is to inhibit cell proliferation and promote cell apoptosis and cycle arrest [34]. FoxO1 is expressed in brain, skeletal muscle, liver, and islet B cells. Most importantly, High level of FoxO1 are expressed in follicular GCs in different develop stages and peak in atresia follicles [35, 36]. Previous studies have found that FoxO1 was associated with PCOS development. It played an important role in promoting follicular atresia and apoptosis of GCs [18, 37]. Unphosphorylated FoxO1 is concentrated in the nucleus of GCs, which enhances the transcriptional activation of downstream apoptotic genes such as p27^kipl^ and Bim [38], thus promoting the apoptosis of GCs. FoxOs is regulated by phosphorylation, acetylation, and proteolysis. The phosphorylation of three highly conserved phosphorylation sites Thr24, Ser256, and Ser319 by PI3K/Akt is the main activity regulation mode [39]. Moreover, it was also confirmed that GLP-1 analogue liraglutide could inhibit apoptosis of pancreatic beta-cell and improve its function through P13K/AKT/FoxO1 pathway [19]. Did GLP-1 affect the proliferation and apoptosis of granulosa cells by modifying the phosphorylation site of FoxO1? As we know, when FoxOs undergo phosphorylation, its nuclear localization signal will be blocked, inducing FoxOs transfer from nucleus to cytoplasm, thus terminating its binding with promoter of downstream target gene [40]. The phosphorylation of ser256 is a prerequisite and necessary condition for the regulation of phosphorylation of other sites. Thr24 can bind to 14-3-3 protein to block the binding of FoxOs to the promoter. After phosphorylation of ser319, it can generate nuclear signal to promote the transfer out of nucleus [41]. Therefore, we chose to mutate the ser256 site of FoxO1 to carry out the subsequent detection. In this study, we found that GLP-1 (7-36) can increase the phosphorylation of ser256 and ser319 sites, promote the proliferation of MGCs, and reduce their apoptosis without inducing the expression of FoxO1 in DHEA-induced PCOS ovarian MGCs. Furthermore, we found that GLP-1 (7-36) could not phosphorylate Foxo1 (Ser 256) effectively after changing the amino acid sequence of Foxo1 (Ser 256) phosphorylation site, nor can it effectively inhibit the apoptosis of GCs. Our data demonstrated that GLP-1 could significantly decrease GCs apoptosis and/or follicular atresia in PCOS mouse ovary. Most importantly, the positive therapeutic effect of GLP-1 on apoptosis in MGCs was associated with phosphorylation modification of FoxO1 expression. Next, we plan to validate GLP-R knockout mice on ovarian granulosa cells to further confirm the effect of GLP-1/GLP-1R axis on the function of granular cells in polycystic ovary mice.

## 5. Conclusion

Overall, our study suggested that the GLP-1/GLP-1R axis acts on PCOS ovarian MGCs to promote their viability *in vivo* and *in vitro*, thereby contributing to oocyte maturation in PCOS. The enhancement of PCOS ovarian MGCs proliferation and antiapoptotic by GLP-1 is mediated, at least partially, by modification of forkhead box protein O1 phosphorylation sites. These findings provided mechanistic insights into the already known beneficial effects of GLP-1RAs on PCOS and supported future efforts to develop more efficient GLP-1RAs for PCOS treatment. Although this study provides a new direction to explore the molecular mechanism of GLP-1RA in the treatment of PCOS, the specific mechanism of GLP-1 modifying FoxO1 site still needs a lot of follow-up research.

## Data Availability

All data used to support the findings of this study are available from the corresponding author upon request.

## Supplementary Materials

Supplementary Materials Figure S1: localization of glucagon-like peptide 1 receptor in mouse ovary. Both immunohistochemical (S1.1) and Western blot (S1.3) analyses were employed to determine the distribution of GLP-1R in mouse ovaries. MGCs were isolated and used confocal microscopy to confirm the localization of GLP-1R in MGCs (S1.2). The immunostaining for the mouse ovarian tissues not only demonstrated the presence of the receptor in mouse ovaries but also identified that the membrane and cytoplasm of oocytes and granulosa cells were the major distribution sites of the receptor in ovaries. The expression of GLP-1R is reduced in PCOS model, and the reduction of GLP-1R in ovarian tissues (F) and granulosa cells (G) in mice with PCOS compared with control mice was tested by qRT PCR (H) and Western blot analysis. Figure S2: HE and FSHR staining was performed with higher-resolution imaging to confirm the identity of granulosa cells (A) Isolated primary granulosa cells from PCOS mice (× 200). (B) After HE staining, the adherent cells showed complete morphology, clear margin, uniform size; large, round nucleus, dark blue, good transparency and rich granules (× 200). (C) Immunofluorescence staining showed FSHR on the cytoplasm was red and nucleus was blue with DAPI staining (× 200). The results confirmed that the isolated cells were granulosa cells of PCOS mouse ovary and the purity of the granulosa cells was over 90%.
