# Supplementary material for: GLP-1/GLP-1R Signaling Regulates Ovarian PCOS-Associated Granulosa Cells Proliferation and Antiapoptosis by Modification of Forkhead Box Protein O1 Phosphorylation Sites
Source: Int J Endocrinol. 2020 Jun 19;2020:1484321. doi: 10.1155/2020/1484321 (PMC7321515; doi:10.1155/2020/1484321)
Supplement: Supplementary Materials — Figure S1: localization of glucagon-like peptide 1 receptor in mouse ovary. Both immunohistochemical (S1.1) and Western blot (S1.3) analyses were employed to determine the distribution of GLP-1R in mouse ovaries. MGCs were isolated and used confocal microscopy to confirm the localization of GLP-1R in MGCs (S1.2). The immunostaining for the mouse ovarian tissues not only demonstrated the presence of the receptor in mouse ovaries but also identified that the membrane and cytoplasm of oocytes and granulosa cells were the major distribution sites of the receptor in ovaries. The expression of GLP-1R is reduced in PCOS model, and the reduction of GLP-1R in ovarian tissues (F) and granulosa cells (G) in mice with PCOS compared with control mice was tested by qRT PCR (H) and Western blot analysis. Figure S2: HE and FSHR staining was performed with higher-resolution imaging to confirm the identity of granulosa cells (A) Isolated primary granulosa cells from PCOS mice (× 200). (B) After HE staining, the adherent cells showed complete morphology, clear margin, uniform size; large, round nucleus, dark blue, good transparency and rich granules (× 200). (C) Immunofluorescence staining showed FSHR on the cytoplasm was red and nucleus was blue with DAPI staining (× 200). The results confirmed that the isolated cells were granulosa cells of PCOS mouse ovary and the purity of the granulosa cells was over 90%. [file 1484321.f1.ppt]

## Slide 1
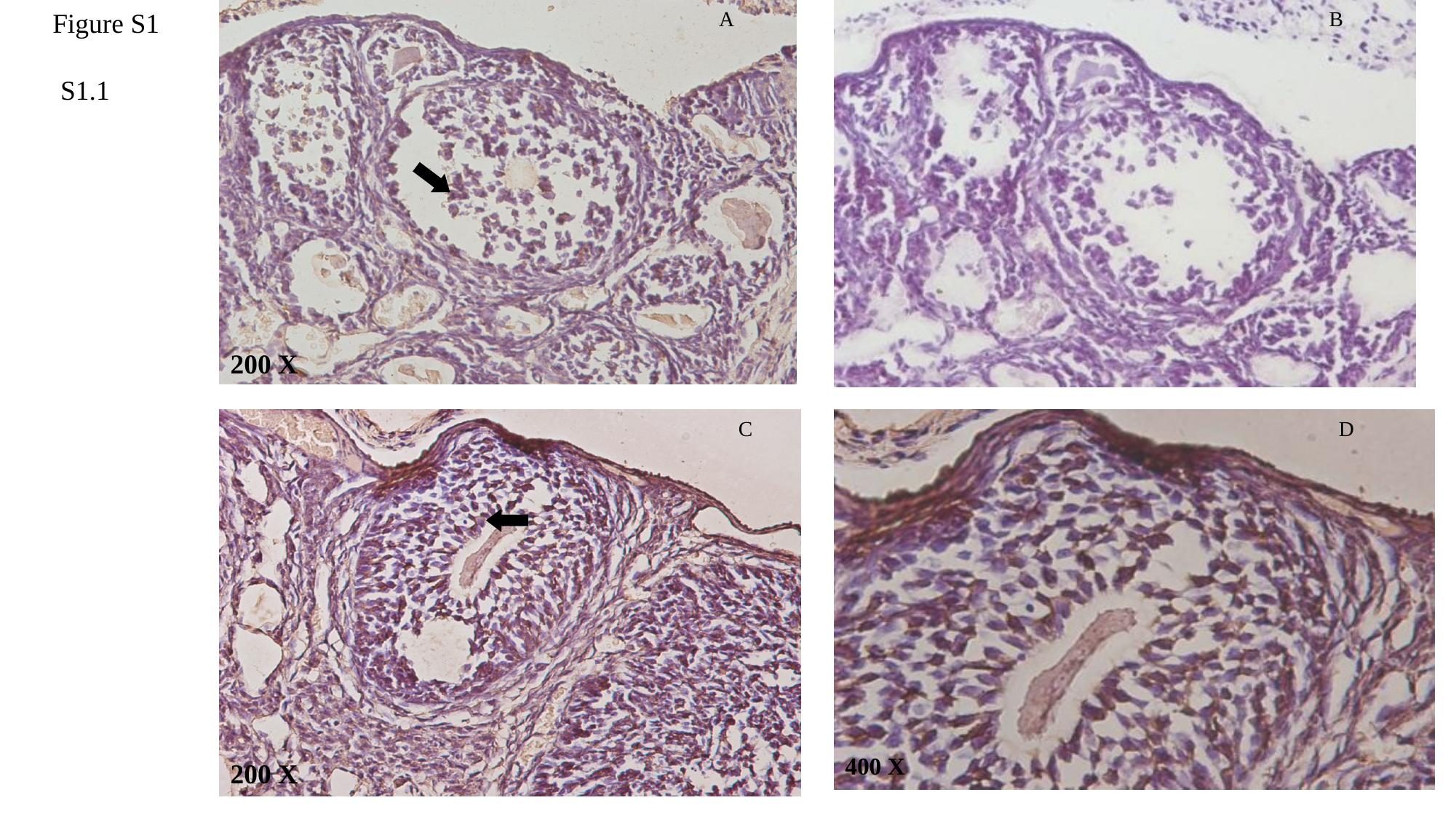

Figure S1
A
B
S1.1
200 X
C
D
400 X
200 X

## Slide 2
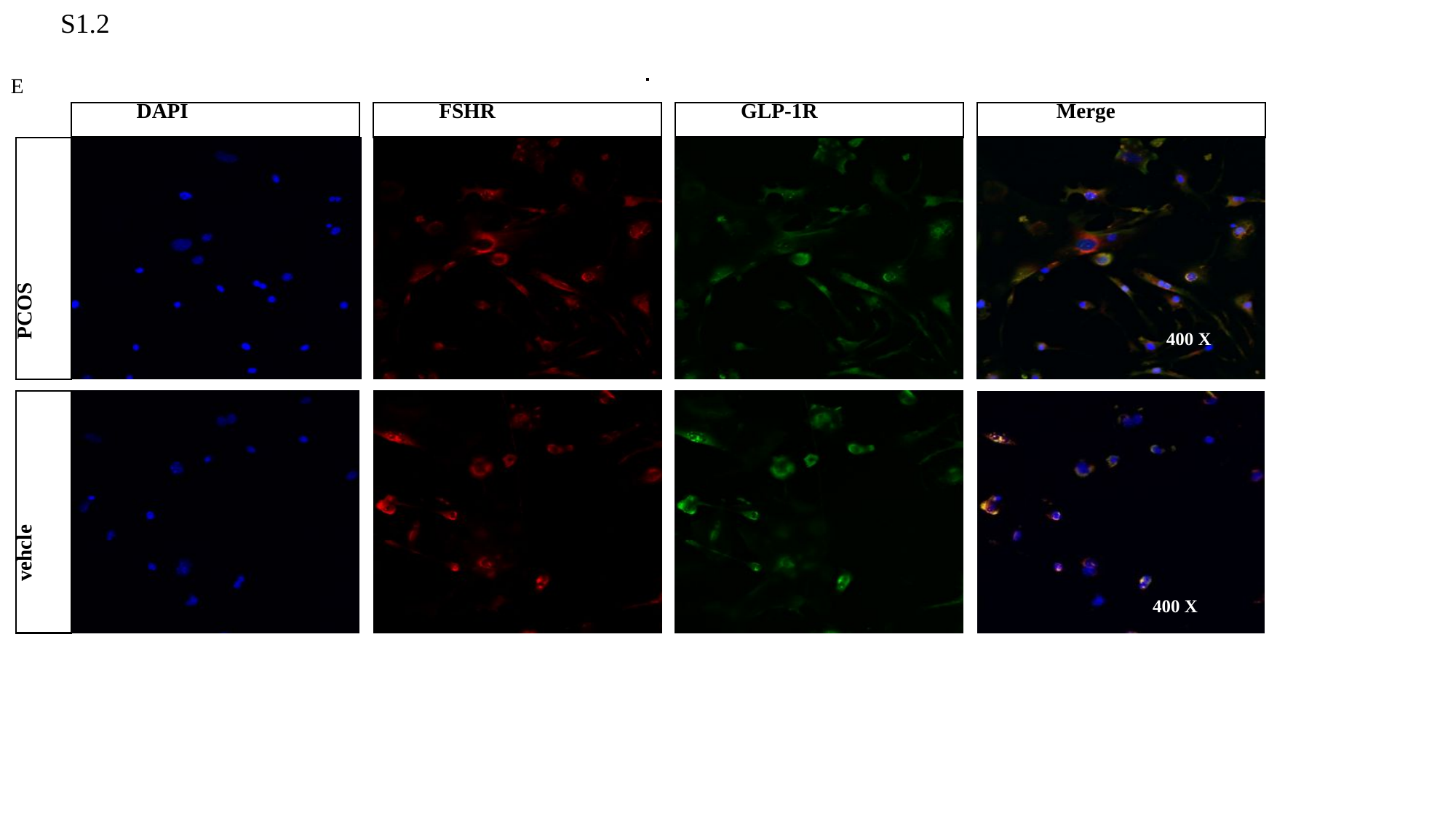

S1.2
E
DAPI
FSHR
GLP-1R
Merge
PCOS
400 X
vehcle
400 X

## Slide 3
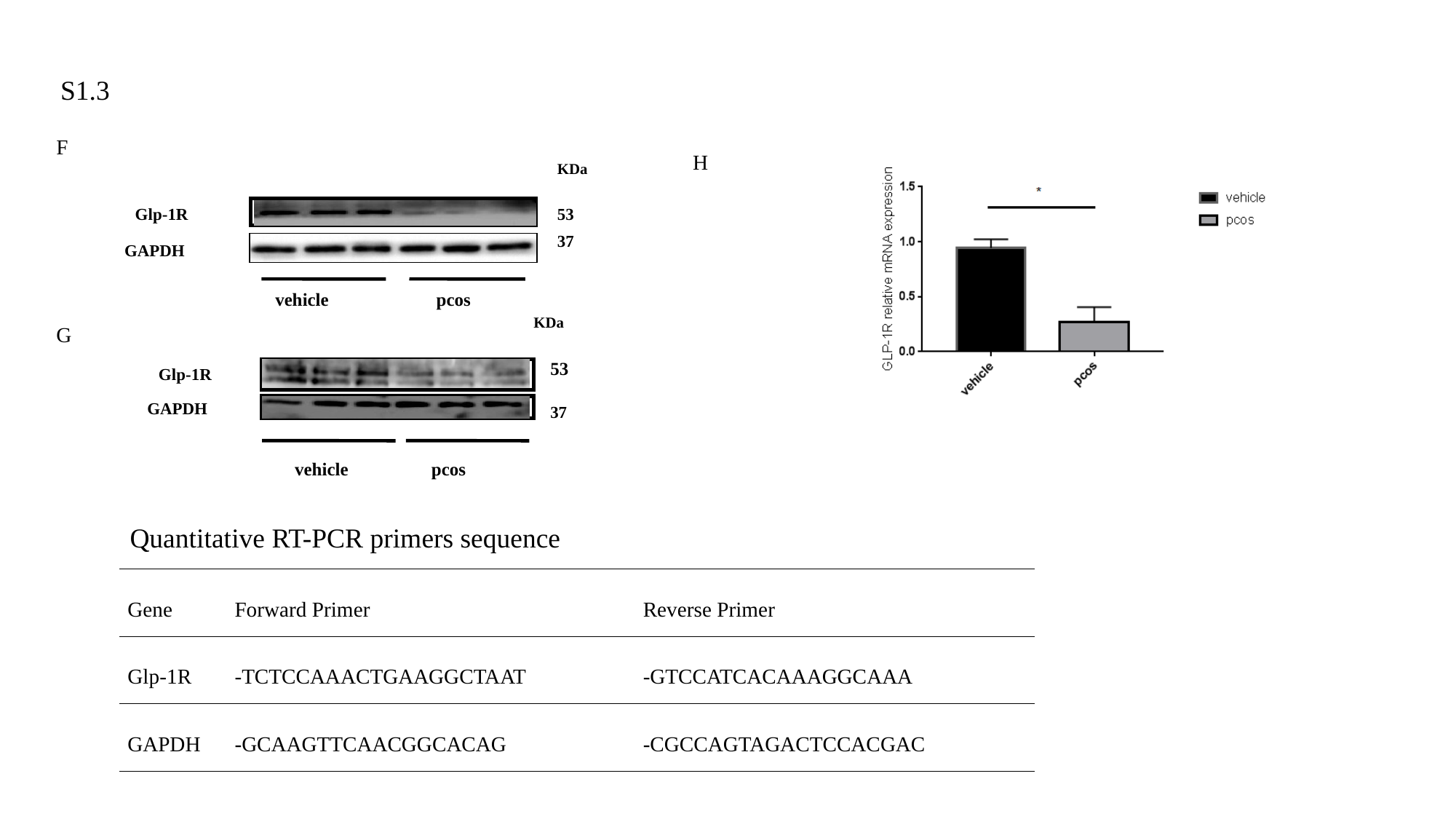

S1.3
F
H
KDa
Glp-1R
53
37
GAPDH
vehicle
pcos
KDa
53
Glp-1R
GAPDH
37
vehicle
pcos
G
Quantitative RT-PCR primers sequence
| Gene | Forward Primer | Reverse Primer |
| --- | --- | --- |
| Glp-1R | -TCTCCAAACTGAAGGCTAAT | -GTCCATCACAAAGGCAAA |
| GAPDH | -GCAAGTTCAACGGCACAG | -CGCCAGTAGACTCCACGAC |

## Slide 4
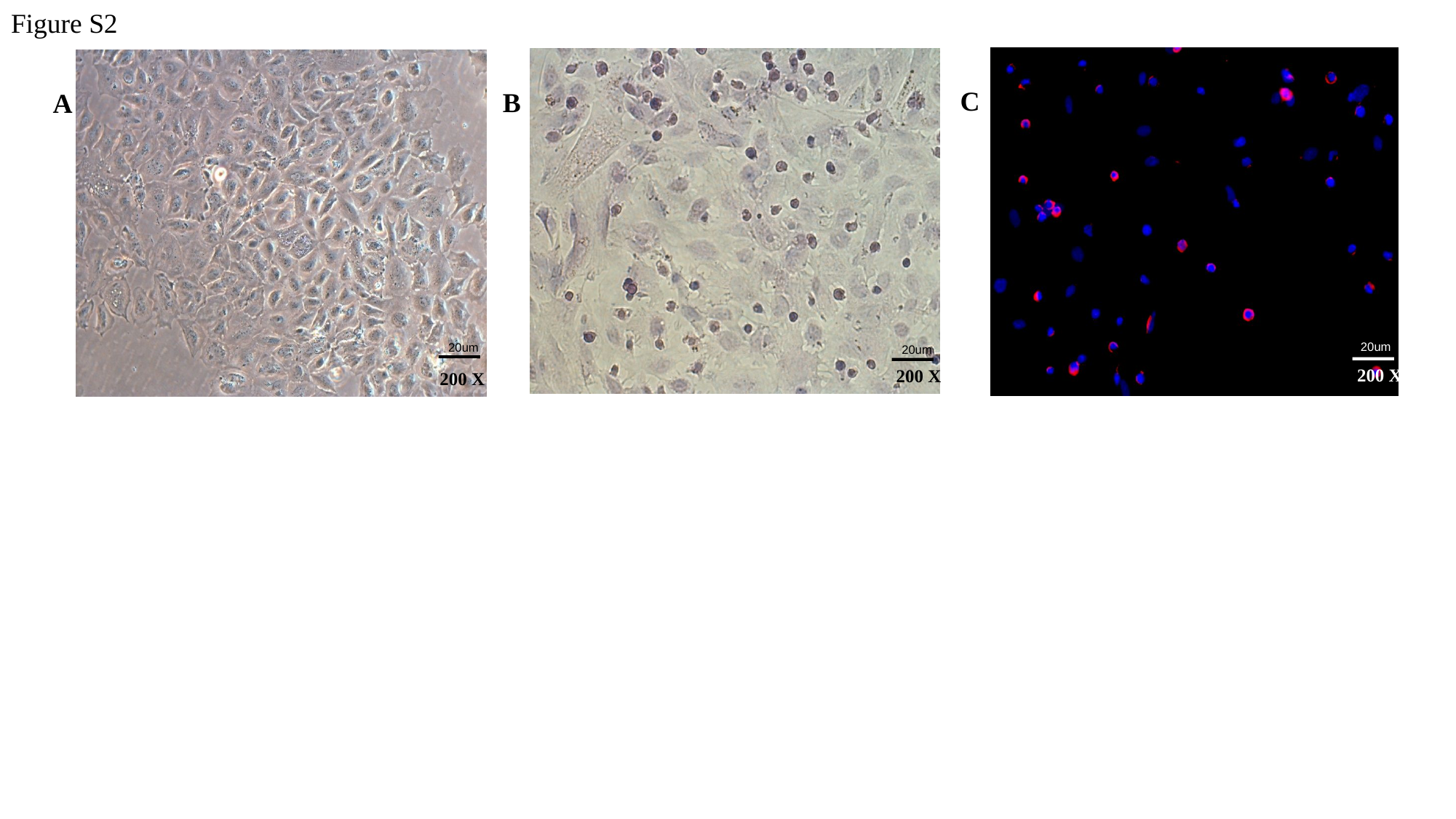

Figure S2
C
B
A
20um
20um
20um
 200 X
 200 X
200 X
